# Electromagnetic fields as structure-function zeitgebers in biological systems: environmental orchestrations of morphogenesis and consciousness

**DOI:** 10.3389/fnint.2014.00084

**Published:** 2014-11-07

**Authors:** Nicolas Rouleau, Blake T. Dotta

**Affiliations:** ^1^Behavioural Neuroscience Program, Laurentian UniversitySudbury, ON, Canada; ^2^Department of Psychology, Laurentian UniversitySudbury, ON, Canada; ^3^Department of Biomolecular Sciences, Laurentian UniversitySudbury, ON, Canada

**Keywords:** electromagnetic fields, consciousness, structure-function, cell aggregation, environmental influences

## Abstract

Within a cell system structure dictates function. Any interaction between cells, or a cell and its environment, has the potential to have long term implications on the function of a given cell and emerging cell aggregates. The structure and function of cells are continuously subjected to modification by electrical and chemical stimuli. However, biological systems are also subjected to an ever-present influence: the electromagnetic (EM) environment. Biological systems have the potential to be influenced by subtle energies which are exchanged at atomic and subatomic scales as EM phenomena. These energy exchanges have the potential to manifest at higher orders of discourse and affect the output (behavior) of a biological system. Here we describe theoretical and experimental evidence of EM influence on cells and the integration of whole systems. Even weak interactions between EM energies and biological systems display the potential to affect a developing system. We suggest the growing literature of EM effects on biological systems has significant implications to the cell and its functional aggregates.

## Introduction

A biological system is dependent upon inter- and intra-cellular communication for its development, maintenance, and proliferation. This communication allows an individual cell to interact with neighboring cell systems as well as its environment. The literature concerning intra- and inter-cellular communication is rapidly growing, focusing on electrical and chemical mechanisms (Qian, 2007; Nielsen et al., 2012; Venturi and Fugua, 2013). However the means by which a biological system can communicate, or interact, through a non-chemical non-electrical medium have yet to be extensively examined. There have been initial studies on the possible contributions of the electromagnetic (EM) spectrum (non-chemical non-electrical) to biological systems (Gurwitsch, 1926; van Wijk et al., 1993; Cifra et al., 2011). These studies have demonstrated that there is, at the very biological systems and the EM spectrum. Here, we look to explore the depth to which surrounding environmental influences, specifically electromagnetic fields (EMF), can have on a developing cell. And more specifically, the effects of EM fields on cell aggregates, whole organisms, and the integration of whole systems.

## Electromagnetic fields (EMF)

Electromagnetic fields (EMF) consist of both electrical and magnetic components manifesting as a field of force. Fields of force can be both static and dynamic. Dynamic fields display temporal variations in intensity that can range from a few cycles per second (Hz) to potentially ~10^43^ cycles per second (Adey, 1980, 1981). The nature of these temporal variations can be symmetrical, such as sine-waves or square waves, or complex, such as the variations generated by cells. The most typical example of the latter would be the action potential of the neuron and the intricately structured dynamics of the cerebral cortical field as inferred by quantitative electroencephalography (QEEG).

The most prominent local magnetic field originates from the Earth. The planet itself generates unique EMFs that have been present since abiogenesis. Paleomagnetic research indicates that polar shifts in the orientation of the Earth’s magnetic field cycle in a ~10^5^ year average periodicity (Banerjee, 2001). On average, its steady-state intensity is about 50,000 nanoTesla (nT). However, there are time-varying changes whose amplitudes are approximately one-thousand times smaller than the steady-state value. There is a large amount of literature showing that these changes in geomagnetic intensity, which are in the order of 10–500 nT, affect biological systems (Adey, 1980, 1981). Most of the contributions to these perturbations originate from the sun and its EM extension: the interplanetary magnetic field or solar wind. However, there are smaller magnitude changes due to lunar orbit and secondary and tertiary effects from inductions of currents within the upper ionosphere. Some of these smaller fluctuations show marked local and diurnal variations. Between the earth and the ionosphere a fundamental frequency (7 to 8 Hz) and its harmonics are generated continuously by what are primarily discharges of lightening (Persinger et al., 2012).

The last century has seen the development of multiple sources of manufactured EMFs added to the natural environment. These manufactured intensities and temporal patterns, through the phenomena of beats (with the subtraction of frequencies the difference emerges as a “virtual” frequency), which have the potential to generate patterns that are so identical to molecular and cellular systems that resonant interactions could occur. The intensities of the manufactured EMFs cross several orders of magnitude. There are now plausible models which indicate that DNA itself behaves as an aggregate of EM antennae that could discern, differentiate, and transform EM energies to differences in base-pair sequences (Cosic, 1994; Mihai et al., 2014). All considered, the EM environment that constitutes the medium in which biological systems are immersed at all times, is highly heterogeneous while operating within the frequency and amplitude bandwidths which are optimally suited to interact with cells.

## Cells and EMFs

Biological systems, from the molecules within the cell, to the cell as an integrate entity, to aggregates of cells that compose organs and organisms, are immersed in a complex mixture of static and time-varying magnetic fields. There are an abundance of hypotheses of how the cell interacts with EMFs. Living cells have a disparity in charge across the plasma cell membrane. Put simply, a living system is not at equilibrium—biological homeostasis involves imbalance. Moving charge across a voltage gradient, or ions through membrane bound channels, would be associated with an EMF, albeit at a weak intensity. However, in an organ such as the brain at any given moment there may be a million cells (10^6^) firing coherently to produce a certain behavior (Levy et al., 2004). This cohesive neuronal firing of 10^6^ cells would produce a substantially larger EMF than any one individual cell.

In addition, other cellular elements like microtubules are thought to play a part in the interaction between EMF and the cell (Havelka and Cifra, 2009; Cifra et al., 2011; Havelka et al., 2011). Microtubules are a component of the cytoskeleton and are comprised of tubulin. In general, these cylindrical polymers average 25 nm in diameter and 100 nm in length. Microtubules are highly involved with cell shape, movement, and growth. They also have the potential to organize functions within the cell (including the neuron) (Gu et al., 2008; Mandal et al., 2011). The tubulin proteins that comprise microtubules are composed of alpha and beta monomers, which produce dipoles resulting in ferroelectric properties (Tuszynski et al., 1995). Because of this, microtubules are highly polar (electrically) structures that allow for the production of EMF. (Hideg et al., 1991; Tuszynski et al., 1995).

In addition to this, exposure to magnetic fields can induce effects in microtubule organization (Glade and Tabony, 2005). Only brief exposure to magnetic fields triggered self-organization within the mitochondria which is central to many cell functions. This elegant experiment demonstrated that the application of an external magnetic field can interact/interfere with biological processes through microtubule self-organization. Despite this mitochondrial self-organization, a cell as a detector of EMFs is much less characterized than EMF production from the cell. There is however research discussing cells as EMF “sensors” (Berzhanskaya et al., 1995, 1996; Potenza et al., 2004). These initial studies discuss bacteria as an organism that is particularly responsive to geomagnetic disturbances. It was found that luminous bacteria displayed increased ultraweak photon emission (UPE) at least 24 h prior to a geomagnetic storm. Furthermore, Berzhanskaya et al. (1995) found that artificially induced applied magnetic fields had an effect on the luminous bacteria’s photon emission as well. They found that only specific frequencies (36–55 GHz), adjacent to fundamental water absorption bands, were successful in altering the bacteria’s photon emission.

It is important to note that only specific frequencies affected the bacteria. Because only select frequencies were successful in varying photon emissions, it implies that there must be a frequency modulated pattern to successfully affect the cells. In other words, a sophisticated “lock and key” system may be the best analogy to describe EMF detection of cells where the fundamental frequency modulated pattern is the “key”. The logical corollary of this proposition is that a given biological system can be functionally described by the sum of its “locks” wherein control of the system is contingent upon the activation of clusters of lock-units to elicit function.

The fact that luminous bacteria reliably and consistently responded to geomagnetic disturbances 24 h prior to the incident suggests they are responding to a non-chemical, non-electrical form of stimuli that was produced before the geomagnetic event took place. Responding to an event before it occurs is not uncommon in the scientific literature. For example, our group (Persinger et al., 2012) found that 2 weeks prior to a geographically distant, magnitude 9 earthquake there were perturbations in the local background photon emissions from Sudbury Ontario. It should be noted that pre-emptive responses to stimuli or events which occur subsequent to the response can be due to the reception of antecedent, unidentified stimuli. In other words, whereas it might seem as though the effects precede the cause, it is possible that a string of events are occurring of which only a select few have been identified. The observed background perturbations in photon emissions which preceded the earthquake might operate as a function of this type of pattern.

These perturbations were noted on 2 separate occasions and produced persistent (~10 day) elevations in background photon emissions. The peak elevations occurred within 24 h of the seismic event. Following the large event there was a consistent drop in background photon emissions that took roughly 10 days to return to normal levels. This example illustrates an energy (photon) increase in a non-local space before an event at a spatially separated location occurred. If a seemingly unrelated process of measuring background photon emissions in the laboratory can be indicative of an impending magnitude >8 earthquakes ~9,000 km away, then a biological system (bacteria) that is sensitive to weak EM perturbations could naturally respond to early EM perturbations of much lesser intensity at potentially comparable distances.

Other cell systems, like mammalian cells, also possess the potential for EM interaction. For example, the depolarization of a neuron produces a massive influx of Calcium (Ca^2+^) through the membrane. This depolarization and flow of ions will produce a low intensity magnetic field. This magnetic field could be mimicked to produce the depolarization of the cells as if the cell had naturally fired (Grassi et al., 2004; Pall, 2013). If a physiologically-patterned, intensity-adjusted EMF was applied to the membrane with the exact resonant frequency of Ca^2+^ gated channels, then this EMF application could in theory activate or stimulate the cell.

## Light and biological systems

A photon is a discrete packet of energy that can be considered a particle-wave. Its energy is directly related to its wavelength and can be calculated with the following equation:

(1) E=hc/λ

Where “*E*” is energy, “*h*” is Planck’s constant, “*c*” is the speed of light, and “*λ*” is wavelength. Photons in the UV to IR range (~200 nm to 1500 nm) have energies ranging from ~6×10^−19^ J–~2×10^−19^ J respectively. Higher energy photons have the potential to affect surrounding tissues (Popp, 1979). The interaction of photons and surrounding tissues could occur as the photons behave as quantum particles that interact via EM forces with other quantum particles in matter (Gabrielli et al., 2006).

Several papers and experiments have shown that virtually all living systems display some level of photon emission (Popp, 1979; van Wijk and van Wijk, 2005). Spontaneous UPE from biological systems has been quantified by Popp to be anywhere from 10^6^–10^7^ photons·s^−1^m^−2^ of tissue. Biological systems emit photons as a component of multiple chemical processes from (very likely) many sources and mechanisms.

Typically, UPE is a product of the chemical reactions of oxygen, specifically reactive oxygen species (Popp, 1979; Rastogi and Pospísil, 2011). Through experimentation, UPE intensity was shown to be highly dependent upon oxygen levels within the system (Tilbury and Quickenden, 1988; Hideg et al., 1991). In fact, under anaerobic conditions there is virtually no UPE from traditional generators. However it should be noted that reactions of oxygen are not the only source of UPE from cells. With any metabolic reaction there will be the subsequent release of a photon. The ensuing photon release can be a marker for activity. In addition, when an electron jumps from one orbit to another there is the release of a photon. The production, role, and theoretical possibilities associated with UPE have significant implications for biomolecular mechanisms.

Biophoton emissions have been recorded from cell cultures (van Wijk et al., 1993; Dotta et al., 2011a), human subjects (Dotta et al., 2011b; van Wijk and van Wijk, 2005), brain slices (Kobayashi et al., 1999), and bacteria (Quickenden, 1974; Tilbury and Quickenden, 1988). Tilbury and Quickenden (1988) have measured photon emissions from *Escherichia coli* (*E. Coli*) during different stages of the growth cycle. In addition to describing photon emission patterns from *E. Coli* during growth phases, they found different frequencies of photons associated with different phases of the growth cycle. The UV (210–310 nm) spectrum contributed most significantly, along with portions of the visible spectrum (450–620 nm), during the exponential growth phase of the bacteria. During the second component of the growth phase (stationary) only the visible region contributed to UPE. The described time and spectral composition of UPE implies a serial order of biophoton emission during division or cellular process. These results also lend support to Cosic’s Resonant Recognition Model for Macromolecules (Cosic, 1994). This model states that specific macromolecules are associated with an optimal spectrum. If specific cellular processes are associated with specific frequency emission, as found by Tilbury and Quickenden (1988), it may be a product of protein turnover within the cell and demonstrate the Cosic model.

The mitochondria are well-known structures within the cell that are thought to produce UPE (Hideg et al., 1991; Kobayashi et al., 1999). The mitochondria play a major role in many cell functions including: energy production, growth, aging, and even communication. Work by Hideg et al. showed copious amounts of UPE from mitochondria of a spinach leaf. Mitochondria induced photon emission was abolished with the administration of inhibitors like antimycin-A and increased by the drug 1,4-diazabicyclo(2,2,2)-octane (DABCO). Given the effectiveness of these drugs it was deduced that singlet oxygen species play an important role in UPE production from mitochondria.

In addition to this, Work by Wu and Persinger (2011) have shown that light irradiation within IR (880 nm) band can increase cell mobility and stem cell proliferation rate in planaria. Studies by Choi et al. (2012) also found that a conformational change in mammalian cells could be induced with visible light (710 nm) irradiation. Choi’s results indicated an increase in MAP-Kinase (MAPK) activity, and subsequent neurite outgrowth in rat cortical neurons, following an ischemic insult only if there was irradiation of 710 nm light. These two studies demonstrate measurable responses of cells and organisms to induced light application. These responses illustrate that light can potentially influence a biological system.

All of the above work on UPE from cells and metabolic reactions demonstrate how common biophoton emissions are within and between living systems. Within and outside the cell there may be a consistent bath of photon radiation distributed across infrared, visible, and ultraviolet wavelengths that interact with neighboring cells. One important question involves the extent, that is, the ultimate impact, from which photons from one cell can influence the photons and hence the associated biomolecular responses in another cell.

## Water and EMFs

Experimental exposures to EMF and accompanying dimensional analyses indicate that information as EM energy can be stored within discrete volumes of water (Gang et al., 2012). Recent experimental evidence confirms the “space-memory” capacities of water wherein pH shifts were altered as a function of pre-exposures to weak-intensity EMF in spring water (Dotta et al., 2013). Additionally, it was found that water pre-exposed to 16 G EMF non-linearly influenced mobility rates and diffusion velocities in planarian worms (Gang and Persinger, 2011). If water can encode and store packets of information—that is, a physical set of instructions or code which is stored and can be potentially accessed for practical use—as energy, and cells are largely composed of water, the implication is that cells can encode and store EM-based information. Furthermore, the observation that chemical reactions are subject to fluctuations corresponding to solar cycles (Piccardi and Capel-Boute, 1972) indicates the potential for variables within the scale of the solar system to influence physical-chemical paradigms. Together, these findings suggest that the local EM environment as the geomagnetic field, which is known to be perturbed by the solar wind, could potentially modulate cellular activity and drive biological structure-function processes.

## Patterned EMFs as structure-function creodes

**Figure 1:**
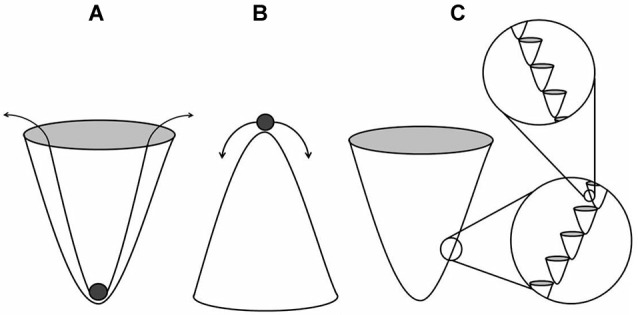
**Stable (A) and unstable (B) states within the landscape of creodes which describe guided influence of morphology**. The deeper or more canalized troughs require more energy to shift the state to an alternative groove within the landscape. Fractal-like grooves **(C)** result in compounded influence.

The mechanisms by which structures are acquired and maintained in biological systems are likely subject to the influence of EMF of the types described previously. An EM basis of morphogenesis has been an ongoing investigation (Burr, 1941; Levin, 2003). A heterogeneous function such as the regulation of morphogenesis requires an analogously heterogeneous substrate from which to draw information. It is currently assumed that a genetic blueprint with modification is largely sufficient in fulfilling this requirement. C.H. Waddington’s epigenetic landscape metaphor was originally employed as a method of describing developmental gene regulation of this sort (Waddington, 1942). A landscape of peaks and troughs (i.e., creodes) is described wherein the geometry of the curved substratum determines the stability of an object within it. An object placed within a trough of extreme depth (Figure 1A) can be considered quite stable wherein a relatively large energy source is required to shift the object to an alternative groove within the landscape. Conversely, an object resting upon a narrow peak can be described as largely unstable (Figure 1B), wherein low energy sources are sufficient to disturb it. This continuum illustrates the limits of biological determinism as a relative concept. It is consistent with the theory to suggest that within a given trough, there would be a fractal-like series of peaks and troughs (Figure 1C), each contributing to a compounding, guided influence of morphology. A recent quantification of cellular differentiation within these landscapes demonstrates the utility of this model (Wang et al., 2011). Stochastic resonance is certainly one way in which a sufficiently noisy background can amplify signals associated with basic cellular processes (Benzi et al., 1981). The stability of a biological system would be determined by whether or not the units were in phase with the background signals.

Suppose that local EMFs operated as functional landscapes of the type described here. The expectation would be that a filtration of the surrounding EM environment or the application of an additional EMF would interfere with the underlying, homeostatic landscape of influence. Constructive and destructive interference of EM peaks and troughs would alter the fundamental structure of the cell or even organism. As is the case in epigenetic modification, subtle energies applied at critical moments of development would be necessary to perturb the system; the phenomenon of teratogenicity would be an example of an outcome expected of this process. In order to observe these effects, the operating landscape must influence a sufficiently large group of cells so as to alter the shape of the organism proper. Perinatal exposures to patterned EMFs induce changes in the morphology of rats (St-Pierre et al., 2007, 2008)—a potential indicator of EM pattern interference. It can also be said that EMFs are able to interfere with the regenerative capacities of the planarian worm (Murugan et al., 2013). In both cases, the systems under influence are under rapid mitotic proliferation and undergoing the fundamental process of cellular differentiation. Under these conditions, the state of the system is inherently transitional and perhaps most vulnerable to perturbations in the EM environment. Based upon these observations, it can be inferred that certain biological states are susceptible to the influence of EMFs which supports the notion that the principles underlying the maintenance of morphology are essentially EM or can, at minimum, interact with the EM spectrum, including visible light. Accepting this line of reason does not negate the effects of genetic influence nor does it imply that EMFs are the unitary force governing morphogenesis. The concept of a morphogenetic field has been previously described (Sheldrake, 2009) and supported in the literature (Mahlberg, 1987; Sheldrake, 1992) which characterizes a pervasive field of influence which guides structure-function. The physical mechanism underlying the influence of morphogenetic fields has remained largely uncharacterized; however, it is clear that EMFs express many of the hypothetically described properties of the morphogenetic field such as an ability to store (Persinger, 2008; Gang et al., 2012) and transmit information (Dotta et al., 2009; Burke et al., 2013). The most common iterations of this phenomenon being modern telecommunication, as well as influence over members of a common morphology (Persinger, 2013). For these reasons, it can be said that the existence of an EM zeitgeber capable of ordering morphogenesis is supported in the literature.

## Redundant patterns in nature and EMFs

The geomagnetic field has been proposed to be a unifying medium in which the immersed biological structures can share functional properties. Persinger (2008) quantified the potential for extracerebral sources of information stored within this medium while suggesting retrieval might involve a reconfiguration of the memory-encoding organ itself (i.e., the hippocampus) within the brain. This emphasis on the requirement of like-structure when attempting to reconstruct information represented in EM form echoes the basic principles of morphogenetic resonance as discussed previously.

A clear redundancy in genomic information is present across species. This is sensible as any effects resulting from genetic-EM interactions would be essentially redundant. As an omnipresent variable on the planet and potential mediator of biological structure formation, the local geomagnetic environment would be expected to generate redundant effects due to the shared genetic variance within and between species. One such reiterative phenomenon that might be expected of this other than common organism geometry is that of critical threshold values involving organized systems (Detrain and Deneubourg, 2006). Social insects are often compared to neuronal structure and function. Honey bee and typical ant colonies consist of approximately 10^4^ members respectively (Beckers et al., 1989; Camazine, 1991), each member of which possesses a brain consisting of ~10^6^ neurons (Menzel and Giurfa, 2001; Detrain and Deneubourg, 2006). Therefore, the number of neurons within a typical honey bee or ant colony is within the same order of magnitude as neurons within the typical human brain or ~10^10^ units/system (Pakkenberg and Gundersen, 1997). Computational models of ants ordering themselves based upon pheromone trails spatially resemble the typical dendritic branching patterns of neurons in the human brain (Detrain and Deneubourg, 2006) and even the fundamental process of synaptic pruning (Chialvo and Millonas, 1995). The accretion of a set number of units within a system is currently assumed to be self-organizing in nature—an emergent property of the complex interactions occurring between the collective of units. The alternative hypothesis is that some hitherto unidentified variable is driving the phenomenon from some external source. One possibility is that information pertaining to nervous system development is partially stored in the extracellular, EM environment. Accessing this information would allow a developing organism to order itself into the appropriate structural configuration required to resonate best with the established archetype contained within the EM engram. Repeating values and critical thresholds would, in this case, be a consequence of a looped line of code being expressed in multiple systems.

## Brain, consciousness, and EMFs

There are biological functions involving cells which have eluded scientific characterization under the traditional biomolecular paradigm. One such example is that of a capacity for the representation of consciousness within a cell or a group of cells. Currently, the scientific study of consciousness relies upon a detailed investigation into the neural correlates of consciousness, where neurons themselves are thought to generate consciousness (Crick and Koch, 1990; Llinas et al., 1998). Penrose and Hameroff (2011) discuss gamma synchrony (30 Hz–90 Hz), the best measurable correlate of consciousness, as not deriving from neuronal firing. The proposed mechanism is that of quantum computation in microtubules with tublin as qubits which is assumed to generate consciousness. The premise that brain function is sufficient to express consciousness must be examined. William James (1898) proposed a transmission theory of consciousness wherein the brain or mind was a passive system or, in principle, a consciousness receiver. Like a radio receiver, the system would require an external signal, the identification of which would clarify the underlying mechanism. Under the assumption that consciousness is an emergent property of complex computation, it is reasonable to characterize every neuroanatomical pathway in search of connectivity thresholds sufficient to generate consciousness. However, if the brain is an aggregate of cells that can passively filter EM energies, the neuroanatomical characterization of the brain will serve only as an epiphenomenal description of structural parameters within which these photo-transductions can occur.

Romijn (2002) proposed that virtual photons—the constituent units of an electromagnetic field—are the essential carrier units of consciousness. Others have suggested zero-energy tachyons (Hari, 2008), or hypothetical particles (Eccles, 1992), both of which are assumed to imbue the biological substrate with the quality of consciousness. In order to define the parameters within which a consciousness carrier particle might operate, energy (E) for 40 Hz oscillations—a neural correlate of consciousness—is obtained by the following equation:

(2) E=hf

Where “*E*” is energy, “*h*” is Planck’s constant, and “*f*” is frequency. The associated energy of a photon oscillating at 40 Hz is 2.65 × 10^−32^ J with a mass equivalency (E = mc²) of 2.94 × 10^−49^ kg. This value constitutes a threshold energy bordering rest-mass of a photon or ~10^−52^ kg (Tu et al., 2005). The implication is that perhaps this value represents the point at which EM carrier particles, departed from rest-mass, can be expressed by the brain as consciousness zeitgebers or psychons with a 40 Hz QEEG correlate. This discrete increase in mass by 10^−50^ kg or energy by ~10^−34^ J (kg m^2^/s^2^) is of potential interest considering that when it is multiplied by a unit of time (s) or divided by a frequency (1/s) it would yield a joule-second or the unit associated with Planck’s constant or ~6.63 × 10^−34^ Js (m^2^kg/s). Planck’s constant describes the quantal units of energy into which the microscopic world is partitioned or the unit which, when multiplied by a frequency, describes the energy of the photon. This discrete unit suggests an EM basis to consciousness at a quantized level. Additionally, the quotient obtained by dividing the computed mass of the 40 Hz psychon by the rest mass of a photon is 10^3^ or a typical order of magnitude within a biological system (e.g., the difference between a cell’s soma and membrane widths). Perhaps it is across this thin boundary that fluctuations in energy can generate photons of sufficient wavelength and associated frequency to achieve the criteria described here. The microtubule quantum computation theory, which offers a potential mechanism for an emergent consciousness, appears highly complex whereas the suggestion here is that the brain can be a passive receiver of light and EM energy. Microtubules, however, continue to represent ideal candidates for a basic receptive unit involved in EM transmission within (and perhaps between) biological systems. In the case of the brain, its relation to consciousness, and its hypothesized EM-receptive capacities, it is not outside the realm of possibilities that a reciprocating signal such as biophoton emissions would be emitted from the cell in order to interact with any external EM zeitgeber.

## Discussion

We have described examples of how energies within the EM spectrum could influence, and potentially be stored within a biological system. The amalgamation of all these results demonstrates the pervasiveness of EM energies across multiple levels of discourse. This unique and important property of EM energies shows its potential as the homogenizer or link across several levels of discourse. Even assuming an interaction of EM energies at a single level, the cell for example, the consequences could be manifested at much higher levels of organization. For example, a magnetic field’s influence on microtubule self-assembly will intervene with biological processes like growth and cell differentiation. This will affect the development of an individual cell. If this magnetic field were to affect a cluster of cells, then effects at higher levels of organization and discourse could develop. Photons are unique in that as they gain or lose energy, their wavelength or spatial occupation is altered. This can be interpreted as a type of inter-dimensional space travel in which a unit of energy can interact with receptive structures along multiple levels of discourse. In pursuit of further knowledge with regard to any subject, paradigm shifts are often necessary to accommodate discoveries which appear to conflict with current models. One shift might involve considering physical, chemical, biological, and social systems to be inextricably connected within the EM environment.

Under the single assumption that biological systems are subject to the influence of EM stimuli found in the external environment, it is reasonable to explore the EM environment for signs of EM signals which display intrinsic organizational features which might inform the areas of morphogenesis or consciousness research. The identification of EM phenomena external to a biological system which appear to generate oscillatory patterns characteristic of physiological activity measured at the level of the cell or even the brain would support the hypothesis that cells are antennae of sorts which resonate with their environments. It has been hypothesized that perhaps biorhythms of the human brain are influenced by resonant oscillations of Earth’s ionisphere: the Schumann resonance (Cherry, 2002). Fundamental biological functions such as blood pressure (Mitsutake et al., 2005) have been linked to Schumann resonance and quantitative solutions converge upon the geomagnetic field as a critical factor involved in consciousness as a fundamental, orchestrating rhythm of brain activity (Persinger et al., 2013). The implication is that an array of rhythms similar to the Schumann-type are perhaps ordering biological activity and orchestrating events dependent upon receptive structures within cells. Perhaps the receptive structures are not only organelles or components of the cytoskeleton proper, but instead the very presence of water. In the balance of probabilities, it is likely that both interactions contribute to a net effect. The complexity of the EM environment will determine its capacity to resonate with the various building blocks which constitute the cell.

The fundamental principles underlying resonance involve synchronous activity when like-structures are exposed to a common stimulus. Departures from identical structure in two systems result in a reduced capacity to resonate. It follows that departing from subatomic organization, the structural homogeneity between and within species will rapidly decrease. For example, all life forms on Earth share the carbon base. Fewer life forms share complete overlap in molecular profiles (i.e., genetic structure). Fewer still share complete or near-complete overlap in proportion of cells expressed resulting in tissue and organ formation. This departure from structural homogeneity is evident by diversity in behaviors observed within and between life forms if structure dictates function. Complex, patterned EM fields optimally influence cells when the application parameters—as temporal organization—are suited to resonate with structures that give rise to the cell including subatomic units (Persinger and Koren, 2007). It stands to reason that a pattern which unifies all or many levels of organization—from the subatomic to the organic and beyond—would provide a fundamental array of signals capable of inducing resonance across multiple levels of discourse. A cell which is subject to a series of EM signals targeting individual proteins, the cytoskeleton, the membrane, and the cell itself would be functionally under control of multiple contributing resonant sources. The landscape of creodes described previously illustrates this environment.

Pathophysiology, under this paradigm, is a consequence of an altered filter. The signal, distorted by a change in structure, is unable to resonate with the system which interferes with normal activity. Although it might seem inconsequential to operate under these assumptions even if they were true, consider approaches in medicine which aim to normalize pathological states. Under the current paradigm, it is necessary to invasively alter the structure of the system itself by surgical or chemical means so as to normalize the function. These protocols invariably result in iatrogenic complications. If EM zeitgebers as resonant phenomena provide input signals that are filtered by the system which ultimately generate functional states—subject to pathological alteration by changes in structure—an alternative approach to the treatment of pathology can involve bending the external signal to suite the current system. In other words, a damaged receiver might not be able to clearly process an external signal; however, with slight modification of the signal, the receiver may be able to process the pre-filtered information and return to a normalized functional state. These virtual-structures would allow for normalized functional states in the absence of the organic tissues typically involved.

Suppose consciousness was to the brain as radio waves are to radio receivers. Identifying the structural and functional qualities of the radio receiver can be very informative when in search of the invisible radio waves; the hardware defines at least some subset of the receptive parameters and exclusionary criteria. In pursuit of the origin of the sounds which seem to originate from within the machine, the scientist employs two main methods of investigation. First, he or she attempts to measure the electrical conduction between parts within the machine, carefully examining every piece of hardware in search of the origin of the signal. Second, he or she attempts to remove components manually or study the function of machines which have incurred damage. Without an understanding of the radio wave, sounds and voices emanating from a radio receiver might appear to be emergent of the complexity of the machine. However, once the radio wave is identified, the system can be studied as a passive piece of hardware—its manifest structure-function. The claim here is that the brain is, at least in part, a passive organ which can receive exogenous EM information and does so naturally. The hypothesized principle outlined here reiterates the basic transmission theories of consciousness. The inevitable extension of this hypothesis is that sources for the neural correlates of consciousness exist outside of the brain or as a physical unit which induces consciousness upon interaction with the brain. In this case, personal consciousness involves reference to a set of memories which can ultimately be subtracted from experiences entirely. Without reference to a set of experiences encoded as memories within the brain, consciousness is uniform or homogeneous. The assumption that consciousness resides only in the brain is based upon an absence of evidence which can never be treated as evidence of absence. This is a testable hypothesis which will involve identifying physical forces outside of the brain which are appropriately patterned to induce the neural correlates of consciousness or experimentally manipulating said pattern to alter consciousness in humans and other animals. As is the case in modern communications technologies, resonance frequencies would be critically important. The physical mechanism of resonance differs from mechanisms associated with technologies such as transcranial magnetic stimulation (TMS) in that, in the case of the former, the patterns of the stimuli rather than the intensity determine the response of the organ.

The postulate presented here is that the cell is merely a place-holder for individuality where a thin, fragile boundary condition is at constant odds with total entropic dissolution. An organ such as the brain can be a passive receiver—a radio with dissolving antennae; it is a structure whose distinct activity slowly fades to static. The implicated physiological machinery operates within an earshot of the subatomic plane. The organ itself is a neighbor to the quantum world, not dominated or restricted to it, but perturbed by the subtle energies that are exchanged at atomic and subatomic scales as EM phenomena. We hypothesize that the structure and function of cells are subject to modification by an ever-present influence: the EM environment. Consciousness, although a cellular phenomenon, does not solely emerge as a function of inordinate complex computations or otherwise convoluted means but is instead a consequence of cellular filtration of light and other components of the EM spectrum.

Experiments can be designed which would confirm this hypothesis. Assuming the EM-zeitgeber model proposed here is correct, there are characteristics of the human brain which imbue it with receptive capacities such that a subset of its function is due to external EM influence. Using abiological materials which express some, but not all of the structural characteristics of the brain and exposing them to the EM environment might reveal intrinsic oscillatory features which are observed in the living, conscious brain. If abiological or non-living, fixed biological materials can express electrical oscillations which are reliably correlated with states of consciousness as inferred by QEEG, the model proposed here will have been supported. One aim must be to separate biological systems from the zeitgebers which order their activity. If the activity observed at the level of the brain or the cell is discovered within the external EM environment using methods of spectral analysis, resonance as discussed here could be occurring. Alternatively, the use of Faraday cages or light-filtering materials, partially insulating biological systems from the EM environment, might sufficiently disrupt fundamental processes of morphogenesis and the neural correlates of consciousness. Simultaneous monitoring of background EM while conducting these experiments will further demonstrate coherence between activity expressed within the immediate environment and within the EM-immersed biological system.

## Conflict of interest statement

The authors declare that the research was conducted in the absence of any commercial or financial relationships that could be construed as a potential conflict of interest.
